# Prevalence and severity of pain, anxiety, stress, and sleep disturbances among surgical patients: a nationwide single-day multicentre flash mob study

**DOI:** 10.1093/bjs/znaf124

**Published:** 2025-07-17

**Authors:** Jetske M Stoop, Roos Geensen, Sophie C Adam, Kayleigh A M van Dam, Els van Dessel, Annemarie Dolmans-Zwartjes, Margot Heijmans, Audrey C H M Jongen, Mirjam Kaijser, Chantal A ten Kate, Joanna Luttikhold, Flores M Metz, Laura van Zeggeren, PASS-Flash Mob Collaborator Group, Antonia S Becker, Isabel Berger, Annelotte Beutler, Lok Sam Samantha Cheng, Manon Bindels, Marco Dam, Thomas L A Dirven, Raphael M J Fischer, Marleen Goddrie, Manuel A Gonçalves Garcia, Tanneke Herklots, Jens Homan, Ellaha Kakar, Elize W Lockhorst, Joost Nonner, Nuray Onayli-Altin, Arno Oomen, Esther van de Poll, Niels Schep, Thomas Schok, D J A Sonneveld, J M E Stam, Joline Stolk, Tamara Remijn-Rentmeester, Natali S Talukder, Sarah Vandenhaute, Emy van der Valk Bouman, Jorrit G Verhoeven, Jacqueline E M Vernooij, Marieke A Vlek, Maud Voesten, José H Volders, Koen J Vree Egberts, Patrick W H E Vriens, Selina J Wijbenga, Wilhelmina A M van Wijngaarden, Helma Zanders, Johannes Jeekel, Markus Klimek

**Affiliations:** Department of Neuroscience, Erasmus Medical Centre, Rotterdam, The Netherlands; Department of Neuroscience, Erasmus Medical Centre, Rotterdam, The Netherlands; Department of Surgery, Reinier de Graaf Gasthuis, Delft, The Netherlands; Department of Surgery, Zuyderland Medical Centre, Heerlen, The Netherlands; Department of Surgery, ZorgSaam ZeeuwsVlaanderen, Terneuzen, The Netherlands; Department of Surgery, Elkerliek Ziekenhuis, Helmond, The Netherlands; Department of Surgery, Máxima Medical Centre, Veldhoven, The Netherlands; Department of Vascular Surgery, Catharina Hospital, Eindhoven, The Netherlands; Department of Surgery, Medisch Centrum Leeuwarden, Leeuwarden, The Netherlands; Department of Surgery, Franciscus Gasthuis & Vlietland, Rotterdam, The Netherlands; Department of Surgery, Ziekenhuis Amstelland, Amstelveen, The Netherlands; Department of Vascular Surgery, Medisch Spectrum Twente, Enschede, The Netherlands; Multi-Modality Medical Imaging Group, TechMed Centre, University of Twente, Enschede, The Netherlands; Dutch Expert Centre for Gastrointestinal Ischaemia, Enschede, The Netherlands; Department of Anaesthesiology and Pain Medicine, Rijnstate Hospital, Arnhem, The Netherlands; Department of Neuroscience, Erasmus Medical Centre, Rotterdam, The Netherlands; Department of Anaesthesiology, Erasmus Medical Centre, Rotterdam, The Netherlands

## Abstract

**Background:**

Patient-reported outcomes (PROs) are subjective health indicators including pain, anxiety, stress, and sleep disturbances. Despite their frequent occurrence in the perioperative period and potentially severe consequences for postoperative recovery (for example prolonged length of hospital stay, cardiovascular events, development of chronic pain), these are not acknowledged as complications and their exact prevalence remains unclear. This study aims to assess the prevalence and severity of pre- and postoperative pain, anxiety, stress, and sleep disturbances among surgical patients.

**Methods:**

A nationwide single-day multicentre cross-sectional flash mob study was conducted in 29 Dutch hospitals. Adult surgical patients with an expected hospital stay of at least one night were included. Patients admitted for neurosurgery, cardiothoracic surgery, or orthopaedic surgery were excluded. Primary outcomes were self-reported pain, anxiety, stress, and sleep disturbances, as assessed with the Numeric Rating Scale, Visual Analogue Scale for Anxiety, Perceived Stress Scale, and the adapted Patient-Reported Outcome Measurement Information System respectively.

**Results:**

Of the 1077 eligible patients, 733 (68%) patients (mean age of 64 ± 15.9 s.d. years, 51.8% male) completed participation. Moderate to severe pain was prevalent in 509 patients (69.7%) and occurred most frequently post-surgery. Anxiety occurred in 278 patients (38.1%) and was more prevalent preoperatively. Moderate to severe stress was reported by 272 patients (37.8%) with similar findings pre- and post-surgery. Sleep disturbances were prevalent in 440 patients (64.1%). Pain and anxiety were more severe in females. Sleep disturbances were more severe in patients with lower socioeconomic status.

**Conclusion:**

Pain, anxiety, stress, and sleep disturbances are highly frequent complications among surgical patients in Dutch hospitals. Considering the prevalence and severity, we suggest implementing these relevant additional measures for PROs as indicators for routine postoperative evaluation to facilitate their management.

## Teaser

A nationwide single-day multicentre cross-sectional flash mob study was conducted in 29 Dutch hospitals to investigate the prevalence and severity of perioperative pain, anxiety, stress, and sleep disturbances among surgical patients. The study showed these are highly frequent complications among surgical patients in Dutch hospitals. Considering the prevalence and severity, we suggest implementing these relevant patient-reported outcome measures as additional indicators for routine postoperative evaluation to facilitate management of these complications.

## Introduction

The five major frequently occurring phenomena during the perioperative period are pain, anxiety, stress, sleep disturbances, and delirium. These may potentially have severe consequences for postoperative recovery, such as increased analgesic and anxiolytic medication use, prolonged length of hospital stay, cardiovascular events, and development of chronic pain^1–3^. Of these, the first four are contributing factors to the development of delirium and can be identified with patient-reported outcome measures (PROMs). Patient-reported outcomes (PROs) are subjective health indicators, as experienced and reported by the patient^4^. Most studies investigating complications and surgical outcomes focus on physiological indicators, such as wound infections, postoperative bleeding, and respiratory failure^5^. The Clavien–Dindo classification captures and grades these surgical complications for both clinical and research applications^6^. However, these PROs are not included in any current classification of complications.

Perioperative pain is linked to higher morbidity rates and lower quality of life impeding swift postoperative recovery, and potentially increasing opioid consumption and medical costs^7^. Patients with preoperative anxiety also report higher levels of postoperative pain and more frequently develop a delirium^8–10^. Moreover, perioperative psychological stress possibly reduces immune responses, elevating the circulating neuroinflammatory mediators, which could lead to an increased risk of postoperative complications including pain and delirium^11–13^. Low postoperative sleep quality is a risk factor for developing delirium and may result in poor recovery due to increased immobility^14,15^.

Currently, the importance of PROs is increasingly acknowledged^16–18^. Although recent research has been performed to further establish the occurrence of pain, anxiety, stress, and sleep disturbances, results are derived from various populations with a variety of outcomes^19,20^. Only few multicentre studies have been conducted within all subspecialties of surgery to obtain a more accurate perspective on the overall prevalence and severity. However, better knowledge of the psychological aspects in the perioperative period could increase the quality of care for these patients.

An appropriate study design for such questions is the flash mob study design, which is a time-efficient method to investigate clinically relevant questions on a large scale^21^. This study aims to establish the prevalence and severity of pre- and postoperative pain, anxiety, stress, and sleep disturbances among surgical patients in the Netherlands on a single day.

## Methods

### Study design

This study was a nationwide, single-day, multicentre cross-sectional flash mob study. All study data were collected on 29 November 2023 between 9 a.m. and 5 p.m. In each participating centre, a team of local researchers (for example residents, nurses, physician assistants), led by the local study coordinator (a surgeon), was assembled to approach eligible patients. After written informed consent was obtained, participants completed a survey consisting of validated questionnaires to assess the primary outcomes. The Erasmus MC Medical Ethics Committee approved this study on 2 October 2023 (MEC-2023-0232).

In a flash mob study design, the sample size is entirely dependent on the time frame of participant inclusion and the number of eligible participants during this time frame. The sample size was therefore not precalculated but aimed at 1000 participants. Assuming an average of 40 available beds per surgical nursing ward, the targeted number of hospitals to participate was at least 25.

### Participants

Patients ≥16 years admitted to the nursing ward of the surgical department of a participating hospital with an expected hospital stay of at least one night were included in the study, including patients who were treated non-operatively. Sufficient knowledge of the Dutch language and ability to assess questionnaires were required for participation. Patients who were unable to give informed consent were excluded. Admission for neurosurgery, cardiothoracic surgery, or orthopaedic surgery was considered an exclusion criterion to specify outcomes on general surgery. For logistic reasons and considering our limited resources, the study was restricted to surgical wards as organized in the Dutch healthcare system. Patients were addressed when treated at the respective departments of General Surgery, including some urology and gynaecology patients.

### Outcome measures

Primary outcomes were pain, anxiety, stress, and sleep disturbances. Pain was assessed using the Numeric Rating Scale (NRS), which is a validated 11-point scale that is widely accepted due to its comprehensibility to patients and its simple intuitive interpretation^22,23^. NRS scores of the worst pain, the average experienced movement-evoked pain, and the average experienced pain at rest during the previous 24 h were collected retrospectively^24^. The severity of pain was classified into the following categories: NRS 1–3 equals mild pain, NRS 4–6 equals moderate pain, and NRS 7–10 equals severe pain^25^.

Anxiety was assessed using the Visual Analogue Scale for Anxiety (VAS-A). The VAS-A is proven to be a valid and effective instrument to assess anxiety^26^. Current level and highest level of anxiety over the previous 24 h in retrospect were measured on a scale between 0 and 100. Anxiety was considered to be present for VAS-A ≥ 34^27^ .

Stress was assessed using the Perceived Stress Scale (PSS-10), which is a 10-item shorter version of the original 14-item Perceived Stress Scale^28^. A Dutch translation of the PSS-10 was used, as previously developed by the Longitudinal Aging Study Amsterdam, according to the principles of good translation^29^. PSS-10 assessed the stress level over the previous month. The total PSS-10 score was calculated by the sum of the score of the negative items and the reverse score of the positive items, so that higher total scores indicate higher stress levels. Low stress was defined as total scores 0–13, moderate stress applied to total scores 14–26, and high stress was defined as total scores 27–40. In addition, the positive items were added up to a perceived self-efficacy score ranging from 0 to 16, for which higher scores indicate higher self-efficacy. The negative items were added up to a perceived helplessness score ranging from 0 to 24, for which higher scores indicate higher helplessness^30^.

Sleep quality was assessed using the adapted Patient-Reported Outcomes Measurements Information System (PROMIS)^31^. This questionnaire subjectively assesses sleep quality using two positive and three negative items on sleep and a sixth item on general experience of sleep quality. The questionnaire assessed sleep quality during the previous night, that is, the night before the planned day of survey conduction. Sleep quality was also assessed over the time period of the month before hospital admission in order to allow for establishing sleep disturbances by comparing home and hospital situations. Similar to the PSS-10, a raw summary score of sleep quality, ranging from 0 to 24, was calculated from the negative score and the reverse positive scores, for which higher summary scores indicate lower sleep quality.

In addition to the primary outcome measures, patient characteristics (for example age, sex), factors related to socioeconomic status (postal code, educational level, monthly income), and characteristics of the surgical procedure were collected, all of which was self-reported. The postal code was matched to the corresponding socioeconomic status (SES-WOA) score from Statistics Netherlands (CBS)^32^. The SES-WOA score is calculated using multiple correspondence analysis based on average financial welfare, educational level, and recent employment history in the postal code area. Educational level was categorized according to the International Standard Classification of Education (ISCED-2011)^33^. Lastly, monthly income was categorized based on the average Dutch net monthly household income in 2022 of EUR 2500^34,35^.

### Statistical analysis

SPSS version 28.0.1.0 was used for statistical analysis. A one-sided *P* < 0.05 was considered statistically significant.

Normality of the variables was assessed based on the Kolmogorov–Smirnov test for large sample sizes and graphically through histograms and QQ-plots. Primary outcomes were reported using descriptive statistics. Prevalence of the primary outcomes was presented as the mean with standard deviation for parametric variables, or the median with interquartile range for non-parametric variables. Severity was presented as the occurrence rate within categories. Sleep disturbances specifically were reported as positive mean differences in the raw summary score, comparing home and hospital sleep quality.

The Mann–Whitney U test was used for testing non-parametric primary outcomes within subgroups with two categories. For subgroups with >2 categories, the Kruskal–Wallis test was used instead. Mean differences in sleep quality between home and hospital situation were compared using Wilcoxon signed-rank test for paired samples. The relationship between SES-WOA and both stress and sleep disturbances was assessed using univariable linear regression.

## Results

All Dutch hospitals were approached for participation in the study, of which 29 of 71 (41%) agreed to participate.

### Participants

**Fig. 1:**
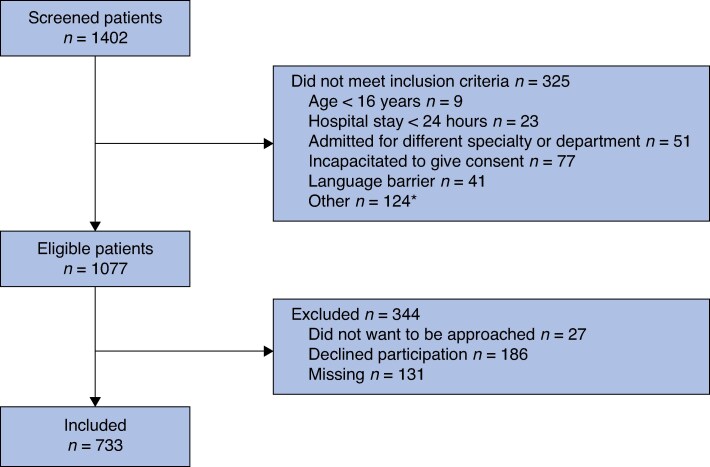
Flow chart *For example isolation, convict, recent return from surgery.

Of the 1077 eligible patients, 733 (68.1%) patients were included in the study (*Fig. 1*). Of all questionnaires, 68 (9.3%) were incomplete, corresponding to 0.8% missing answers in total.

Patient demographics are presented in *Table 1*. Mean age was 64 years (15.9) years and 380 (51.8%) were male. The majority of participants (78.5%) were included post-surgery. The distribution of all parameters was non-parametric.

**Table 1:** Patient demographics

Category	
Study population, *n*	733
Age, mean (SD)	64.3 (15.9)
**Sex**	
Male	380
Female	353
Socioeconomic status (SES-WOA), median (i.q.r.)	0.039 (−0.134–0.166)
**Level of education**	
Low	331 (45.2)
Intermediate	240 (32.7)
High	155 (21.1)
Unknown	7 (1.0)
**Monthly income**	
No income	33 (4.5)
EUR ≤2500	312 (42.5)
EUR 2501–5000	173 (23.6)
EUR ≥5001	18 (2.5)
Unknown	197 (26.9)
**Perioperative period**	
Preoperative	80 (10.9)
Postoperative	575 (78.5)
No surgical procedure planned	78 (10.6)
**Use of sleep medication in past 24 h**	167 (22.8)
**Use of pain medication in past 12 h**	637 (86.8)

Values are *n* (%) unless otherwise indicated. SES-WOA, socioeconomic status based on welfare; educational level and recent employment history.

### Primary outcomes

**Fig. 2:**
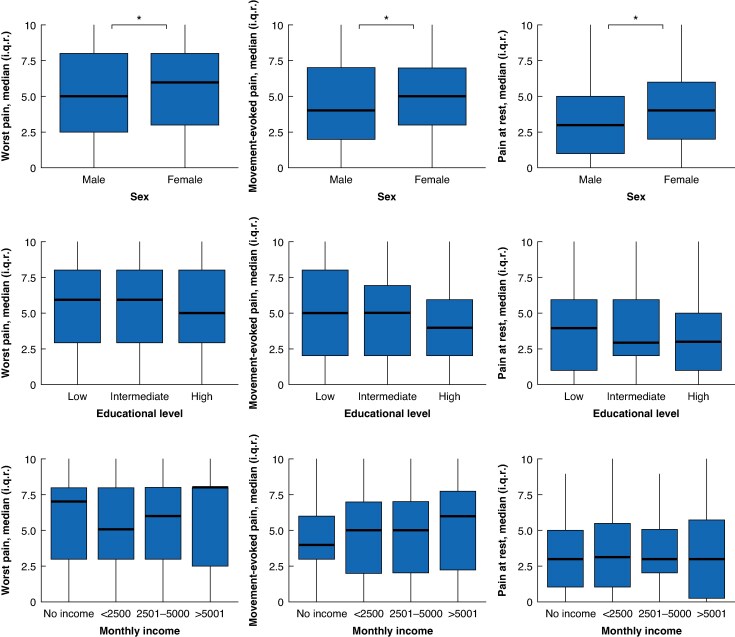
Pain in subgroups of sex, educational level, and monthly income *P < 0.05.

Median score regarding the worst pain from the last 24 h was 6 (3–8), with 509 patients (69.7%) reporting moderate or severe pain during this time (*Table 2*). Absolute pain scores did not significantly differ between pre- and postoperative included participants, but prevalence of pain was highest among postoperative included participants when compared to preoperative inclusions or those with no surgery planned (91.6% *versus* 76.3% *versus* 88.5%, *P* < 0.001). Female patients reported more severe pain (*Fig. 2*, *Table S2*). Regarding educational level and monthly income, no significant differences were found.

**Table 2:** Prevalence and severity of pain in surgical patients

Total group	*N* (%)	Median (i.q.r.)
**Worst pain (*n* = 730)**		6 (3–8)
NRS ≥ 1	654 (89.6)	
Mild pain NRS 1–3	145 (19.9)	
Moderate pain NRS 4–6	184 (25.2)	
Severe pain NRS 7–10	325 (44.5)	
**Movement-evoked pain (*n* = 729)**		5 (2–7)
NRS ≥ 1	646 (88.6)	
Mild pain NRS 1–3	176 (24.1)	
Moderate pain NRS 4–6	225 (30.9)	
Severe pain NRS 7–10	245 (33.6)	
**Pain at rest (*n* = 730)**		3 (1–6)
NRS ≥ 1	605 (82.9)	
Mild pain NRS 1–3	245 (33.6)	
Moderate pain NRS 4–6	231 (31.6)	
Severe pain NRS 7–10	129 (17.7)	

NRS, numeric rating scale.

**Fig. 3:**
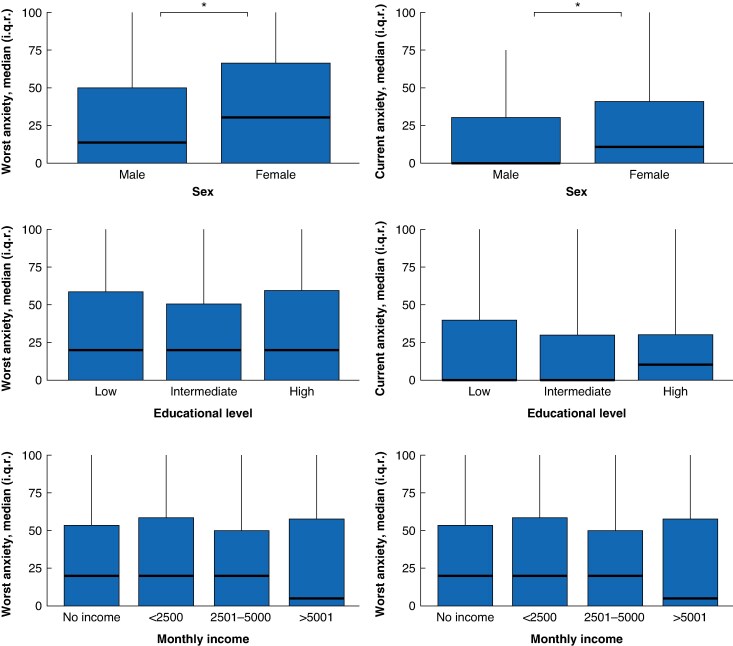
Anxiety in subgroups of sex, educational level, and monthly income *P < 0.05.

Median worst reported anxiety was 20 (0–60) (*Table 3*). Moderate to severe anxiety in the previous 24 h was experienced by 278 patients (38.1%). The prevalence of moderate to severe anxiety was highest in the preoperatively included patients compared to postoperative inclusions or those with no surgery planned (40.0% *versus* 23.1% *versus* 29.5%, *P* = 0.004). Preoperative included patients also reported significantly higher anxiety scores (median 21 (0–60) *versus* 6.5 (0–30) *versus* 0 (0–42), *P* = 0.004) (*Table S1*). Female patients reported higher anxiety scores, but no significant differences were found for educational level and monthly income (*Fig. 3*, *Table S3*).

**Table 3:** Prevalence and severity of anxiety in surgical patients

Total group	*N* = 730
Worst anxiety, median (i.q.r.)	20 (0–60)
Anxiety VAS-A 34–100, *n* (%)	278 (38.1)
Current anxiety, median (i.q.r.)	8 (0–39)
Anxiety VAS-A 34–100, *n* (%)	187 (25.6)

VAS-A, visual analogue scale for anxiety.

**Fig. 4:**
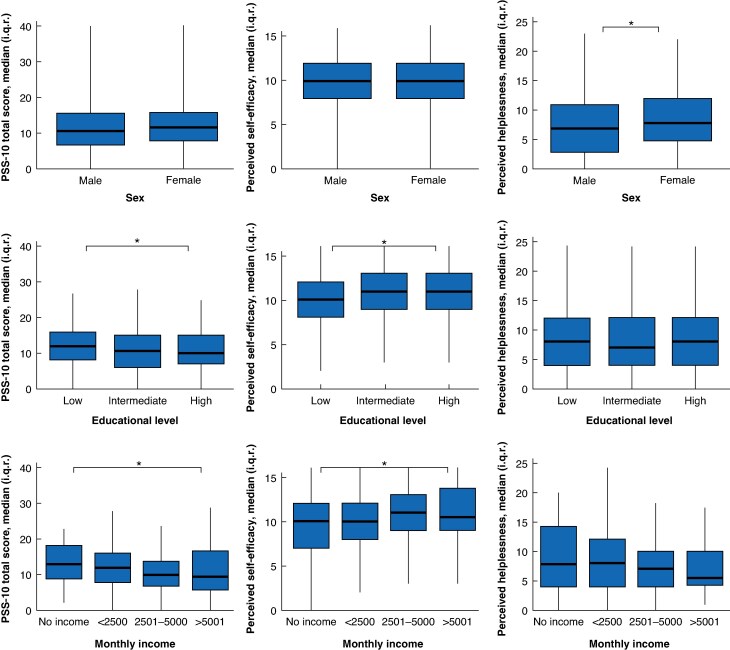
Stress in subgroups of sex, educational level, and monthly income *P < 0.05, PSS-10 = perceived stress scale.

The median (i.q.r.) total stress score was 11 (7–16) (*Table 4*). Moderate to severe stress scores were prevalent in 272 participants (37.8%). Patients perceived self-efficacy with a median of 10 (8–12) of 16, and helplessness with a median of 8 (4–12) of 24. There were no significant differences found between pre- and postoperative perceived stress scores. Participants with low education and low monthly income experienced significantly more stress and perceived themselves less self-efficient (*Fig. 4*, *Table S4*). The linear regression model showed a significant decrease in stress score for an increase in SES-WOA scores (β = −0.364, *P* = 0.009) (*Fig. S1*).

**Table 4:** Prevalence and severity of subjective stress in surgical patients

Total group	*N* (%)	Median (i.q.r.)
**Total PSS-10 score (*n* = 720)**		11 (7–16)
Low stress 0–13	448 (62.2)	
Moderate stress 14–26	264 (36.7)	
High stress 27–40	8 (1.1)	
Perceived self-efficacy (*n* = 721)		10 (8–12)
Perceived helplessness (*n* = 725)		8 (4–12)

PSS-10, perceived stress scale.

**Fig. 5:**
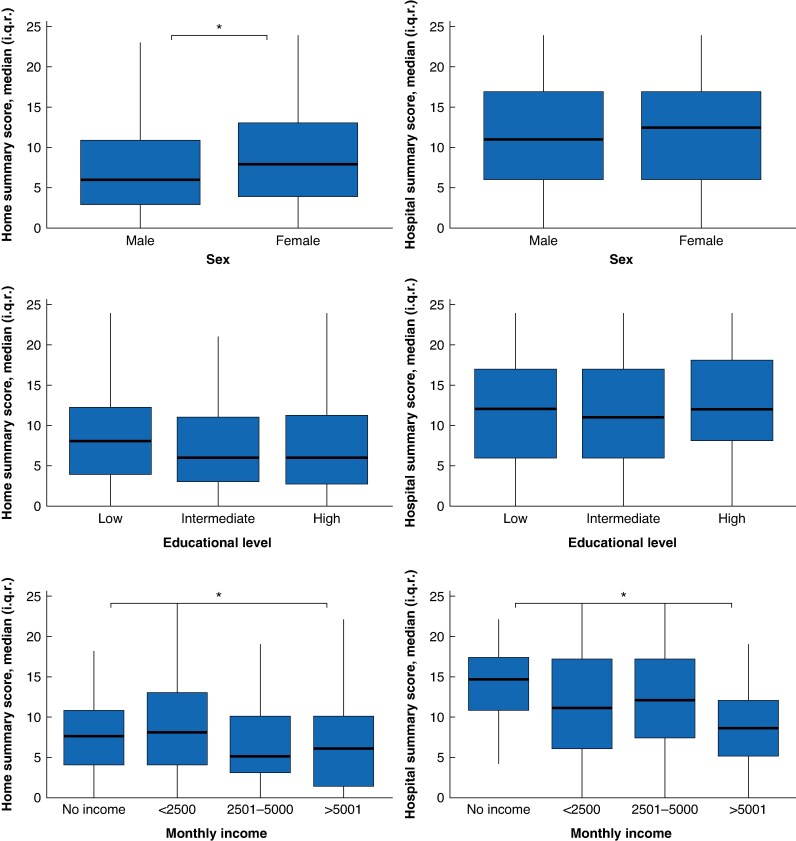
Sleep disturbances in subgroups of sex, educational level, and monthly income *P < 0.05.

The mean difference between sleep quality at home and during hospital admission was 3.68 (95% c.i.: 3.09 to 4.28, *P* < 0.001) (*Table 5*). The number of positive mean differences, indicating the number of participants who experienced worse sleep quality in the hospital compared to at home, was 440 (64.1%). Among patients who had no surgery planned, the prevalence of sleep disturbances was significantly higher, compared to preoperative and postoperative patients respectively 78.9% *versus* 58.7% *versus* 63.0% (*P* = 0.018). Patients in lower income categories experienced lower sleep quality, both at home and during hospital admission (*Fig. 5*, *Table S5*). The mean difference between home and hospital sleep quality was also the largest in this group. Lower SES-WOA scores were also associated with more sleep disturbances (β = 0.377, *P* = 0.05) (*Fig. S2*).

**Table 5:** Prevalence and severity of sleep disturbances in surgical patients

Total group	*n* = 686
Home summary score, median (i.q.r.)	7 (3–12)
Hospital summary score, median (i.q.r.)	11 (6–17)
Mean difference (95% c.i.)	3.68 (3.09–4.28)
Number of positive mean differences, *n* (%)	440 (64.1)

## Discussion

This nationwide multicentre cross-sectional flash mob study showed that in the surgical population, pain was prevalent in 70%, anxiety and stress in 38%, and sleep disturbances in 64%. The prevalence of pain was higher post- rather than preoperatively, whereas anxiety occurred more frequently preoperatively. Sleep disturbances occurred most frequently in patients who did not undergo a surgical procedure. Females reported higher pain and anxiety scores.

The selection of PROs in clinical research can be challenging^36^. The parameters we have chosen here are clinically relevant and assessable by validated instruments. As the results show, they are also very common in the surgical population often accepted as ‘part of the procedure’ and not recognized as preventable and treatable complications. The International Consortium for Health Outcomes Measurement (ICHOM) provides some feasible standard sets for the surgical patient^37^. However, we feel that the standard sets developed are too specific, for example narrowed to colorectal cancer patients, for the diversity of the overall surgical population in this study. Furthermore, the dedicated questionnaires are frequently time-consuming, which was not compatible with the flash mob design^37^. To our knowledge, this was the first large multicentre flash mob study to investigate the prevalence of PROs within the surgical population. More than 40% of all Dutch hospitals participated, including both peripheral and tertiary centres across the Netherlands. Therefore, our study population was highly reflective of the surgical population as a whole, as confirmed by the distribution of socioeconomic indicators in our study population, which was similar to the distribution in the Dutch population^34^.

Other studies using average pain scores in rest per postoperative day suggest that clinically relevant perioperative pain affects approximately 30–65% of patients undergoing a surgical procedure^19,38,39^. In contrast, we used the worst pain experienced in the previous 24 h, which possibly explains our higher prevalence of clinically relevant pain. Using worst pain as outcome parameter better covers the seriousness of pain as a complication. Looking at the prevalence, 87% of patients in our study population received pain medication in the last 24 h. However, looking at the severity, we found that the average pain score at rest was below the treatment threshold of NRS ≤ 3, whereas the worst score was NRS = 6. Therefore, it can be concluded that pain in peak moments is severe, but in general pain is properly managed by pharmacological interventions on the surgical ward.

For preoperative anxiety, wide-ranging prevalence rates between 19% and 93% have been suggested in studies assessing the outcome prior to the pre-anaesthetic visit at the outpatient clinic^8,10,40,41^, while we collected data during admission to the nursing ward. Two of these studies used the Amsterdam Preoperative Anxiety and Information Scale (APAIS) reporting conflicting occurrence rates^8,40^. Using the VAS-A scale, there was a preoperative anxiety rate of 40% and postoperative 38% and, to our knowledge, postoperative anxiety has not been previously studied. Measuring subjective stress is challenging and perception of and reaction to stress is multidimensional with considerable variation both between and within individuals^42^. For subjective stress, in relation to the risk on negative health outcomes, validated questionnaires include, for example, the Subclinical Stress Symptom Questionnaire (SSQ-25), the Stress Overload Scale (SOS), and the PSS-14 or PSS-10^42,43^. With the flash-mob design, the length of the questionnaire had to be brief and thus the PSS-10 was used despite the limitation of lacking validation of the Dutch translated version^29^. Another challenge is the interpretation of the PSS-10 and recent literature established higher validity for a two-factor interpretation of the PSS-10^30^. However, classification of perceived self-efficacy and helplessness has not yet been identified. To retrieve optimal results, we provided both the perceived self-efficacy and helplessness scores, as well as calculated a total summary score for grading severity.

Recent studies demonstrate that hospitalized patients experience sleep deprivation^44,45^. A meta-analysis demonstrated a prevalence of 60% of sleep disturbances in the surgical population, which is in line with our findings^45^. Another flash mob study using the same adapted PROMIS to assess sleep disturbances found a higher sleep quality both during hospital admission and at home than our study, and a smaller decrease in sleep quality during admission compared to home (mean difference 3.0, 95% c.i. 2.7 to 3.4)^31^. However, this study was performed in all hospitalized patients, whereas we specifically looked at the surgical population. The reduction of sleep quality during admission was the largest in the surgical group of the population of that flash mob study, corresponding with our findings. Another meta-analysis among all hospitalized patients demonstrated that sleep duration of 76% of patients is approximately 1–4 h shorter during hospital admission^44^. The fact that patients without a surgical procedure experienced higher sleep disturbances cannot be explained by our data. However, one might hypothesize that not being sure about either the diagnosis or the need of a surgical intervention is more stressing and sleep disturbing than having a diagnosis and undergoing surgery.

Although perioperative pain is generally adequately managed, treatment usually involves the use of opioid analgesics. Opioids are highly effective to control pain, but can also cause serious negative health outcomes, such as unwanted sedation, respiratory depression, prolonged hospital stay, increased medical costs and prolonged chronic opioid consumption, which can lead to addiction or misuse^2,46–49^. In the Netherlands, hospital admissions due to opioid intoxication have tripled, and the number of people treated for opioid addiction has doubled between 2008 and 2014^50^. The other PROs, namely anxiety and sleep disturbances, are commonly treated with benzodiazepines, also causing cognitive adverse effects and increasing the risk of persistent opioid use^2,51^. Although these pharmacological interventions are usually effective, treatment options without the aforementioned negative consequences should be explored. Strategies to reduce opioid use are for example pre-emptive and non-opioid analgesics and regional anaesthesia techniques^52^. Non-pharmacological treatments, such as music intervention, preoperative education and counselling, and Enhanced Recovery After Surgery (ERAS) protocol could be implemented to improve PROs^53–57^.

This study has major limitations. First, all study data were collected through a single questionnaire, depending on the patient’s understanding and recollection measuring subjective outcomes, and there may be recall bias. For the PROs, this was a key strength, but markedly limited the categorization of the procedures because characteristics of the surgical procedure were collected through the questionnaire as free text entry by the patient. Second, data collection was limited to the verbal information provided by the participant to ensure feasibility and efficiency of the study, without highly burdening the patient or the healthcare staff. Therefore, the study design did not allow for collecting information on the type of anaesthesia, or for the specific admission indication for non-operated patients. Also, data on sleep and pain medication might not be entirely accurate. The third limitation was the lower-than-anticipated inclusion rate due to a higher rejection rate and number of missed patients. The fourth limitation was that the flash mob design of this study did not allow for investigating delirium, which is clinically diagnosed based on criteria from the Diagnostic and Statistical Manual of the American Psychiatric Association (DSM-5)^58^ and patients with delirium are incapacitated and unable to provide informed consent. However, the investigated indicators are, in fact, risk factors for delirium^14,59^.

In conclusion, pain, anxiety, stress, and sleep disturbances are highly frequent phenomena among surgical patients in the hospital, occurring in up to 78.9% of patients, and should not be considered unavoidable, but as preventable and treatable complications. Considering the incidence and severity of these complications, we suggest future studies focusing on early preoperative screening and effective prevention and treatment during the perioperative phase.

## Collaborators

Antonia S. Becker (Erasmus Medical Centre, Rotterdam, The Netherlands), Isabel Berger (Maasstad Ziekenhuis, Rotterdam, The Netherlands), Annelotte Beutler (Maasstad Ziekenhuis, Rotterdam, The Netherlands), Lok Sam Samantha Cheng (Erasmus Medical Centre, Rotterdam, The Netherlands), Manon Bindels (Canisius-Wilhelmina Ziekenhuis, Nijmegen, The Netherlands), Marco Dam (Ziekenhuis Nij Smellinghe, Drachten, The Netherlands), Thomas L.A. Dirven (Erasmus Medical Centre, Rotterdam, The Netherlands), Raphael M.J. Fischer (Erasmus Medical Centre, Rotterdam, The Netherlands), Marleen Goddrie (Admiraal de Ruyter Ziekenhuis, Goes, The Netherlands), Manuel A. Gonçalves Garcia (Alrijne Ziekenhuis, Leiderdorp, The Netherlands), Tanneke Herklots (Treant Zorggroep, Emmen, The Netherlands), Jens Homan (Gelre ziekenhuizen, Apeldoorn, The Netherlands), Ellaha Kakar (Maastricht UMC+, Maastricht, The Netherlands), Elize W. Lockhorst (Amphia Ziekenhuis, Breda, The Netherlands), Joost Nonner (Ikazia Ziekenhuis, Rotterdam, The Netherlands), Nuray Onayli-Altin (Bravis Ziekenhuis, Bergen op Zoom, The Netherlands), Arno Oomen (Anna Zorggroep, Geldrop, The Netherlands), Esther van de Poll (Gelre ziekenhuizen, Apeldoorn, The Netherlands), Niels Schep (Maasstad Ziekenhuis, Rotterdam, The Netherlands), Thomas Schok (VieCuri Medical Centre, Venlo, The Netherlands), D.J.A. Sonneveld (Dijklander Ziekenhuis, Hoorn, The Netherlands), J.M.E. Stam (Medisch Spectrum Twente, Enschede, The Netherlands), Joline Stolk (Anna Zorggroep, Geldrop, The Netherlands), Tamara Remijn-Rentmeester (Admiraal de Ruyter Ziekenhuis, Goes, The Netherlands), Natali S. Talukder (Dijklander Ziekenhuis, Hoorn, The Netherlands), Sarah Vandenhaute (ZorgSaam ZeeuwsVlaanderen, Terneuzen, The Netherlands), Emy van der Valk Bouman (Erasmus Medical Centre, Rotterdam, The Netherlands), Jorrit G. Verhoeven (Erasmus Medical Centre, Rotterdam, The Netherlands), Jacqueline E.M. Vernooij (Rijnstate Ziekenhuis, Arnhem, The Netherlands), Marieke A. Vlek (Elisabeth-TweeSteden Ziekenhuis, Tilburg, The Netherlands), Maud Voesten (Ziekenhuis Nij Smellinghe, Drachten, The Netherlands), José H. Volders (Diakonessenhuis, Utrecht, The Netherlands), Koen J. Vree Egberts (Medisch Spectrum Twente, Enschede, The Netherlands), Patrick W.H.E. Vriens (Elisabeth-TweeSteden Ziekenhuis, Tilburg, The Netherlands), Selina J. Wijbenga (Franciscus Gasthuis & Vlietland, Rotterdam, The Netherlands), Wilhelmina A.M. van Wijngaarden (Gelre ziekenhuizen, Apeldoorn, The Netherlands), Helma Zanders (Diakonessenhuis, Utrecht, The Netherlands).

## Supplementary Material

znaf124_Supplementary_Data

## Data Availability

Data and study documents will become available after publication upon reasonable request. Request for data access can be submitted to the corresponding author.
