# Supplementary material for: Prevalence and severity of pain, anxiety, stress, and sleep disturbances among surgical patients: a nationwide single-day multicentre flash mob study
Source: Br J Surg. 2025 Jul 17;112(7):znaf124. doi: 10.1093/bjs/znaf124 (PMC12268324; doi:10.1093/bjs/znaf124)
Supplement: znaf124_Supplementary_Data [file znaf124_supplementary_data.docx]

**Prevalence and severity of Pain, Anxiety, Stress and Sleep disturbances among surgical patients: the PASS-Flash mob study**

**A nation-wide single-day multicentre flash mob study**

Jetske M. Stoop *PhD Candidate*^1^, Roos Geensen *Research Physician*^2^, Sophie C. Adam *Registered Nurse*^3^, Kayleigh A.M. van Dam *Research Physician*^4^, Els van Dessel *Medical Specialist*^5^, Annemarie Dolmans-Zwartjes *Physician Assistant*^6^, Margot Heijmans *Research Coordinator*^7^, Audrey C.H.M. Jongen *Physician*^8^, Mirjam Kaijser *Medical Specialist*^9^, Chantal A. ten Kate *Physician*^10^, Joanna Luttikhold *Medical Specialist*^11^, Flores M. Metz *Research Physician*^12^, Laura van Zeggeren *Physician*^13^, PASS-Flash mob Collaborator Group*, Johannes Jeekel *Professor*^14^, Markus Klimek *Associate Professor*^15^

^1^BSc., Department of Neuroscience, Erasmus Medical Centre, Rotterdam, The Netherlands

^2^MD., Department of Neuroscience, Erasmus Medical Centre, Rotterdam, The Netherlands

^3^MSc., Department of Surgery, Reinier de Graaf Gasthuis, Delft, The Netherlands

^4^MD., Department of Surgery, Zuyderland Medical Centre, Heerlen, The Netherlands

^5^MD., Department of Surgery, ZorgSaam ZeeuwsVlaanderen, Terneuzen, The Netherlands

^6^MSc., Department of Surgery, Elkerliek ziekenhuis, Helmond, The Netherlands

^7^PhD., Department of Surgery, Máxima Medical Centre, Veldhoven, The Netherlands

^8^MD., PhD., Department of Vascular Surgery, Catharina Hospital, Eindhoven

^9^MD., Department of Surgery, Medisch Centrum Leeuwarden, Leeuwarden, The Netherlands

^10^MD., PhD., Department of Surgery, Franciscus Gasthuis & Vlietland, Rotterdam, The Netherlands

^11^MD., PhD., Department of Surgery, Ziekenhuis Amstelland, Amstelveen, The Netherlands

^12^MD., (a) Department of Vascular Surgery, Medisch Spectrum Twente, Enschede, The Netherlands, (b) Multi-Modality Medical Imaging Group, TechMed Centre, University of Twente, Enschede, The Netherlands, (c) Dutch Expert Centre for Gastrointestinal Ischaemia, Enschede, The Netherlands

^13^MD., Department of Anaesthesiology and Pain Medicine, Rijnstate Hospital, Arnhem, The Netherlands

^14^MD., PhD., Department of Neuroscience, Erasmus Medical Centre, Rotterdam, The Netherlands

^15^MD., PhD., Department of Anaesthesiology, Erasmus Medical Centre, Rotterdam, The Netherlands

Corresponding Author; Miss Jetske Stoop (0009-0001-2430-8144), address: Dr. Molewaterplein 40, 3015 GD Rotterdam, email: j.m.stoop@erasmusmc.nl, phone: +31 10 7040704.

**Supplementary Materials - Index**

| **Supplementary Figures and Tables** |  |
| --- | --- |
| Table S1: Median pre- and postoperative pain, anxiety, stress and sleep disturbances. | *page 3* |
| Table S2: Prevalence and severity of pain in surgical patients in subgroups. | *page 4* |
| Table S3: Prevalence and severity of anxiety in surgical patients in subgroups. | *page 5* |
| Table S4: Prevalence and severity of subjective stress in surgical patients in subgroups. | *page 6* |
| Figure S1: The relationship between socioeconomic status (SES-WOA) and stress (PSS-10 total score). | *page 7* |
| Table S5: Prevalence and severity of sleep disturbances in surgical patients in subgroups. | *page 8* |
| Figure S2: The relationship between socioeconomic status (SES-WOA) and sleep disturbances (PROMIS mean difference). | *page 9* |
| Table S6: Surgical procedure planned or performed among participants. | *page 10* |
| Table S7: Primary outcomes in subgroups of type of surgery. | *page 11* |
|  |  |
|  |  |

**Table S1: Median pre- and postoperative pain, anxiety, stress and sleep disturbances.**

|  | **Preoperative, median (IQR)** | **Postoperative, median (IQR)** | **No surgical procedure, median (IQR)** | **p-value** |
| --- | --- | --- | --- | --- |
| **Pain** |  |  |  |  |
| Worst pain | 5 (1-8) | 6 (3-8) | 7 (3-8) | 0.280 |
| Movement-evoked pain | 5 (1.25-7) | 5 (2-7) | 6 (2-8) | 0.720 |
| Pain at rest | 4 (0-6) | 3 (1-5) | 4 (2-7) | 0.156 |
| **Anxiety** |  |  |  |  |
| Worst anxiety | 23.5 (0-60) | 20 (0-60) | 20 (0-49.5) | 0.590 |
| Current anxiety | 21 (0-59.75) | 6.5 (0-30) | 0 (0-42.25) | 0.004* |
| **Stress** |  |  |  |  |
| Total score | 10 (7-16) | 11 (7-16) | 13 (8-17) | 0.212 |
| Perceived self-efficacy | 11 (8-12) | 10 (8-12) | 10 (8-12) | 0.210 |
| Perceived helplessness | 7 (3-12) | 8 (4-12) | 8 (3.5-12.5) | 0.620 |
| **Sleep** |  |  |  |  |
| Home summary score | 6 (4-11.5) | 7 (3-12) | 5 (2.75-10) | 0.270 |
| Hospital summary score | 11 (6-18) | 11 (6-17) | 13.5 (7.25-20) | 0.054 |
| Mean difference (95% CI) | 3.47 (7.31) | 3.36 (7.90) | 6.41 (8.08) | 0.012* |

IQR = interquartile range, CI = confidence interval.

**Table S2: Prevalence and severity of pain in surgical patients in subgroups.**

|  | **By perioperative period, n(%)** | | | | | | |
| --- | --- | --- | --- | --- | --- | --- | --- |
|  | **Preoperative**  (n = 572) | | **Postoperative**  (n = 80) | | **No surgery planned** (n = 78) | | **p-value** |
| **Worst pain** |  | |  | |  | |  |
| NRS ≥ 1 | 61 (76.3) | | 524 (91.6) | | 69 (88.5) | | <0.001* |
| - Mild pain NRS 1-3 | 10 (12.5) | | 121 (21.2) | | 14 (17.9) | |  |
| - Moderate pain NRS 4-6 | 19 (23.8) | | 150 (26.2) | | 15 (19.2) | |  |
| - Severe pain NRS 7-10 | 32 (40.0) | | 253 (44.2) | | 40 (51.3) | |  |
| **Movement-evoked pain** |  | |  | |  | |  |
| NRS ≥ 1 | 64 (80.0) | | 519 (90.7) | | 63 (81.8) | | 0.003* |
| - Mild pain NRS 1-3 | 17 (21.3) | | 147 (25.7) | | 12 (15.6) | |  |
| - Moderate pain NRS 4-6 | 19 (23.8) | | 187 (32.7) | | 19 (24.7) | |  |
| - Severe pain NRS 7-10 | 32 (41.6) | | 185 (32.3) | | 32 (41.6) | |  |
| **Pain at rest** |  | |  | |  | |  |
| NRS ≥ 1 | 58 (72.5) | | 482 (84.3) | | 65 (83.3) | | 0.033* |
| - Mild pain NRS 1-3 | 16 (20.0) | | 209 (36.5) | | 20 (25.6) | |  |
| - Moderate pain NRS 4-6 | 25 (31.3) | | 182 (31.8) | | 24 (30.8) | |  |
| - Severe pain NRS 7-10 | 17 (21.3) | | 91 (15.9) | | 21 (26.9) | |  |
|  | **By sex, median (IQR)** | | | | | | |
|  | **Male** (n = 379) | | | **Female** (n = 349) | | | **p-value** |
| Worst pain | 5 (3-8) | | | 6 (3-8) | | | 0.019* |
| Movement-evoked pain | 4 (2-7) | | | 5 (2.75-7) | | | 0.003* |
| Pain at rest | 3 (1-5) | | | 4 (1-6) | | | 0.005* |
|  | **By educational level, median (IQR)** | | | | | | |
|  | **Low** (n = 329) | | **Intermediate** (n = 239) | | **High** (n = 155) | | **p-value** |
| Worst pain | 6 (2.25-8) | | 6 (3-8) | | 5 (3-8) | | 0.890 |
| Movement-evoked pain | 5 (2-7) | | 5 (2-7) | | 4 (2-6) | | 0.170 |
| Pain at rest | 4 (1-6) | | 3 (2-5) | | 3 (1-5) | | 0.630 |
|  | **By monthly income, median (IQR)** | | | | | | |
|  | **No income** (n = 33) | **EUR ≤2500** (n = 311) | | **EUR 2501-5000** (n = 172) | | **EUR ≥5001** (n = 18) | **p-value** |
| Worst pain | 7 (2.5-8) | 5 (3-8) | | 6 (3-8) | | 8 (1.75-8.25) | 0.600 |
| Movement-evoked pain | 4 (2.5-6) | 5 (2-7) | | 5 (2-7) | | 6 (1.75-8) | 0.670 |
| Pain at rest | 3 (1-5) | 4 (1-6) | | 3 (2-5) | | 3 (0-6) | 0.750 |

IQR = interquartile range, NRS = numeric rating scale.

**Table S3: Prevalence and severity of anxiety in surgical patients in subgroups.**

|  | **By perioperative period, n(%)** | | | | | | |
| --- | --- | --- | --- | --- | --- | --- | --- |
|  | **Preoperative** (n = 80) | | **Postoperative (**n = 572) | | **No surgery planned** (n = 78) | | **p-value** |
| **Worst anxiety** |  | |  | |  | |  |
| Anxiety VAS-A 34-100 | 31 (38.8) | | 220 (38.5) | | 27 (35.1) | | 0.840 |
| **Current anxiety** |  | |  | |  | |  |
| Anxiety VAS-A 34-100 | 32 (40.0) | | 132 (23.1) | | 23 (29.5) | | 0.004* |
|  | **By sex, median (IQR)** | | | | | | |
|  | **Male** (n = 379) | | | **Female** (n = 349) | | | **p-value** |
| Worst anxiety | 13 (0-50) | | | 29.5 (0-66.75) | | | 0.001* |
| Current anxiety | 0 (0-30) | | | 10 (0-40) | | | 0.013* |
|  | **By educational level, median (IQR)** | | | | | | |
|  | **Low** (n = 329) | | **Intermediate** (n = 239) | | **High** (n = 155) | | **p-value** |
| Worst anxiety | 20 (0-59.75) | | 20 (0-51) | | 20 (0-60) | | 0.640 |
| Current anxiety | 0 (0-40) | | 0 (0-30) | | 10 (0-31) | | 0.320 |
|  | **By monthly income, median (IQR)** | | | | | | |
|  | **No income** (n = 33) | **EUR ≤2500** (n = 310) | | **EUR 2501-5000** (n = 173) | | **EUR ≥5001** (n = 18) | **p-value** |
| Worst anxiety | 20 (0-57.25) | 20 (0-59.25) | | 20 (0-50) | | 5 (0-61.75) | 0.830 |
| Current anxiety | 4 (0-39.5) | 9.5 (0-40) | | 10 (0-25.5) | | 0 (0-30.25) | 0.480 |

IQR = interquartile range, VAS-A = visual analogue scale for anxiety.

**Table S4: Prevalence and severity of subjective stress in surgical patients in subgroups.**

|  | **By perioperative period, n(%)** | | | | | | |
| --- | --- | --- | --- | --- | --- | --- | --- |
|  | **Preoperative** (n = 79) | | **Postoperative** (n = 567) | | **No surgery planned** (n = 74) | | **p-value** |
| Total PSS-10 score |  | |  | |  | | 0.240 |
| - Low stress 0-13 | 51 (64.6) | | 358 (63.1) | | 39 (52**.**7) | |  |
| - Moderate stress 14-26 | 27 (34.2) | | 202 (35.6) | | 35 (47.3) | |  |
| - High stress 27-40 | 1 (1.3) | | 7 (1.2) | | 0 (0.0) | |  |
|  | **By sex, median (IQR)** | | | | | | |
|  | **Male** (n = 375) | | | **Female** (n = 344) | | | **p-value** |
| Total PSS-10 score | 11 (7-16) | | | 12 (8-16) | | | 0.073 |
| Perceived self-efficacy | 10 (8-12) | | | 10 (8-12) | | | 0.740 |
| Perceived helplessness | 7 (3-11) | | | 8 (5-12) | | | 0.016* |
|  | **By educational level, median (IQR)** | | | | | | |
|  | **Low** (n = 324) | | **Intermediate** (n = 238) | | **High** (n = 152) | | **p-value** |
| Total PSS-10 score | 12 (8-16) | | 10.5 (6-15) | | 10 (7-15) | | 0.007* |
| Perceived self-efficacy | 10 (8-12) | | 11 (9-13) | | 11 (9-13) | | <0.001* |
| Perceived helplessness | 8 (4-12) | | 7 (4-12) | | 8 (4-12) | | 0.930 |
|  | **By monthly income, median (IQR)** | | | | | | |
|  | **No income** (n = 32) | **EUR ≤2500** (n = 305) | | **EUR 2501-5000** (n = 173) | | **EUR ≥5001** (n = 18) | **p-value** |
| Total PSS-10 score | 13 (8.25-18.75) | 12 (8-16) | | 10 (7-14) | | 9.5 (5-17.5) | 0.002* |
| Perceived self-efficacy | 10 (7-12) | 10 (8-12) | | 11 (9-13) | | 10.5 (9-14.25) | 0.002* |
| Perceived helplessness | 8 (4-14.75) | 8 (4-12) | | 7 (4-10) | | 5.5 (3.75-11.5) | 0.160 |

IQR = interquartile range, PSS-10 = perceived stress scale.


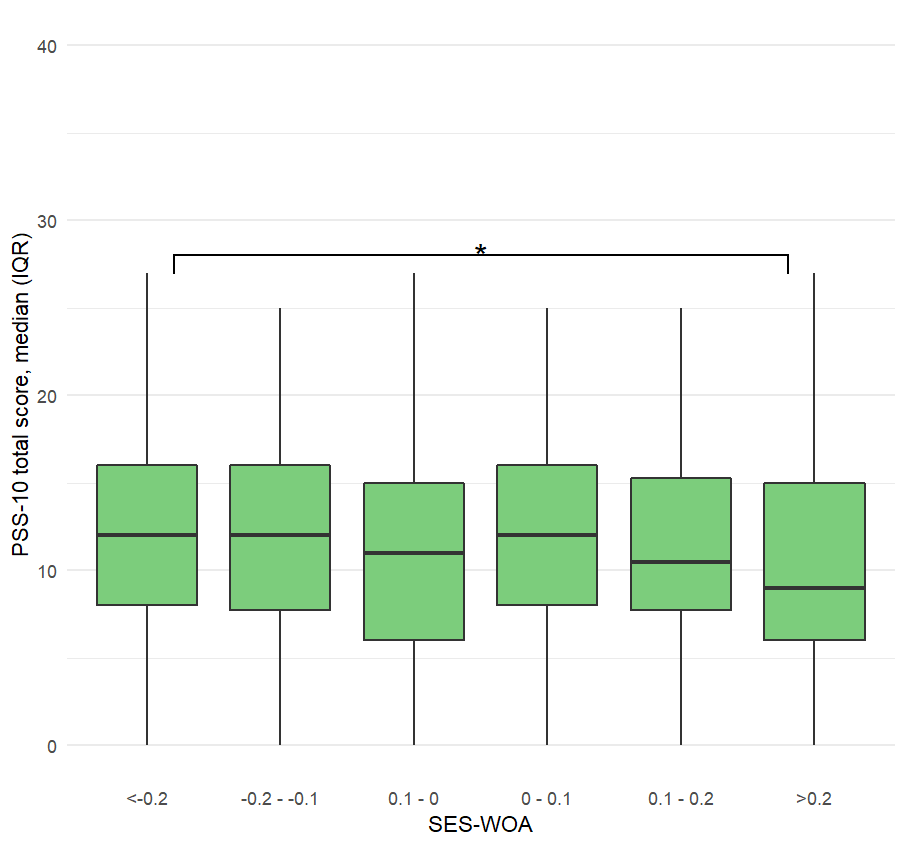


**Figure S1: The relationship between socioeconomic status (SES-WOA) and stress (PSS-10 total score).**

*p = 0.009, PSS-10 = perceived stress scale, SES_WOA = socioeconomic status based on welfare, educational level and recent employment history, IQR = interquartile range.

**Table S5: Prevalence and severity of sleep disturbances in surgical patients in subgroups.**

|  | **By perioperative period** | | | | | | |
| --- | --- | --- | --- | --- | --- | --- | --- |
|  | **Preoperative** (n = 77) | | **Postoperative** (n = 555) | | **No surgery planned** (n = 74) | | **p-value** |
| Home summary score, median (IQR) | 6 (4-11.5) | | 7 (3-12) | | 5 (2.75-10) | | 0.270 |
| Hospital summary score, median (IQR) | 11 (6-18) | | 11 (6-17) | | 13.5 (7.25-20) | | 0.054 |
| Mean difference (95% CI) | 3.47 (7.31) | | 3.36 (7.90) | | 6.41 (8.08) | | 0.012* |
| Number of positive mean differences, n(%) | 44 (58.7) | | 340 (63.0) | | 56 (78.9) | | 0.018* |
|  | **By sex, median (IQR)** | | | | | | |
|  | **Male** (n = 368) | | | **Female** (n = 337) | | | **p-value** |
| Home summary score, median (IQR) | 6 (3-11) | | | 8 (4-13) | | | <0.001* |
| Hospital summary score, median (IQR) | 11 (6-17) | | | 12 (6-17) | | | 0.260 |
| Mean difference (95% CI) | 4.16 (3.36-4.96) | | | 3.16 (2.28-4.04) | | | 0.160 |
|  | **By educational level, median (IQR)** | | | | | | |
|  | **Low** | | **Intermediate** | | **High** | | **p-value** |
| Home summary score, median (IQR) | 8 (4-12) | | 6 (3-11) | | 6 (3-11.75) | | 0.170 |
| Hospital summary score, median (IQR) | 12 (6-17) | | 11 (6-17) | | 12 (8-18) | | 0.460 |
| Mean difference (95% CI) | 2.90 (2.01-3.80) | | 4.03 (3.02-5.03) | | 4.78 (3.46-6.09) | | 0.200 |
|  | **By monthly income, median (IQR)** | | | | | | |
|  | **No income** (n = 30) | **EUR ≤2500** (n = 298) | | **EUR 2501-5000** (n = 169) | | **EUR ≥5001** (n = 18) | **p-value** |
| Home summary score, median (IQR) | 8.5 (4-11) | 8 (4-13) | | 5 (3-10) | | 6 (1-10.75) | <0.001* |
| Hospital summary score, median (IQR) | 14.5 (10.25-17.75) | 11 (6-17) | | 12 (7-17) | | 8.5 (5-12) | 0.047* |
| Mean difference (95% CI) | 5.71 (3.49-7.94) | 2.96 (2.02-3.90) | | 5.90 (4.70-7.09) | | 1.17 (-3.45-5.83) | <0.001* |

IQR = interquartile range, CI = confidence interval.


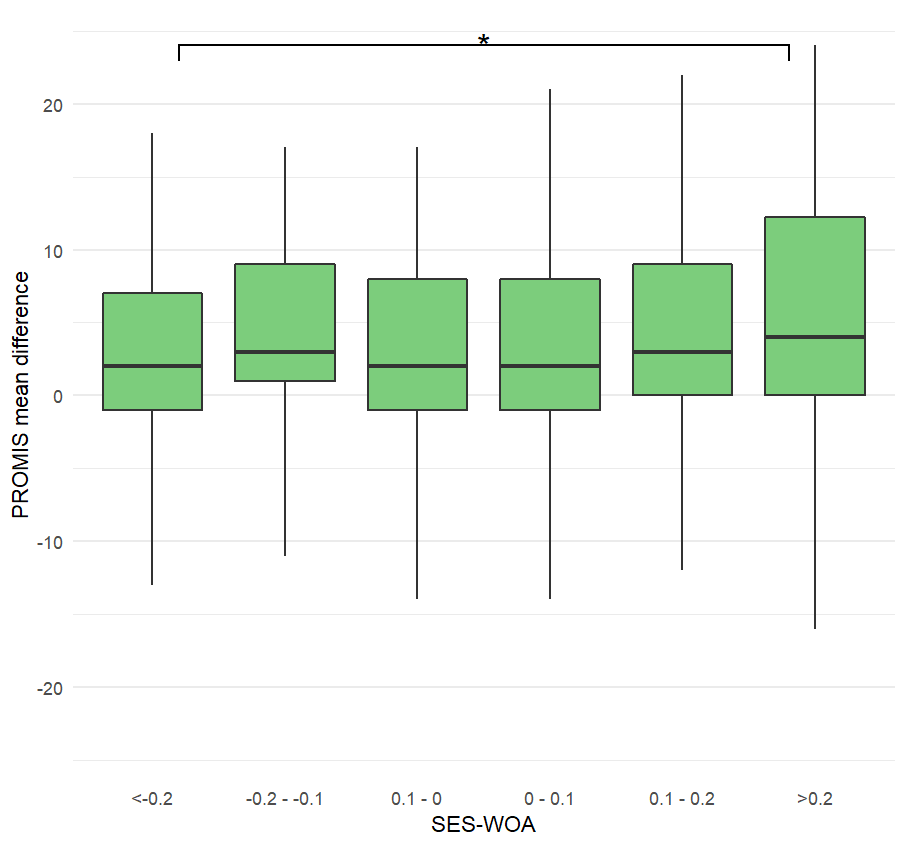


**Figure S2: The relationship between socioeconomic status (SES-WOA) and sleep disturbances (PROMIS mean difference).**

*p = 0.05, PROMIS = Patient-Reported Outcomes Measurement Information System, SES-WOA = socioeconomic status based on welfare, educational level and recent employment history.

**Table S6: Surgical procedure planned or performed among participants.**

| Surgical procedure planned or performed, n(%) | n = 733 |
| --- | --- |
| - Gastro-intestinal | 230 (31.4) |
| - Vascular | 116 (15.8) |
| - Trauma | 92 (12.6) |
| - Oncological* | 64 (8.7) |
| - Urological** | 54 (7.4) |
| - Other^ | 99 (13.5) |
| - No surgical procedure | 78 (10.6) |

*Surgeries were only categorized as oncological if the description clearly specified that it was an oncological procedure, e.g. tumour, cancer.

**Urological non-oncological procedures included surgical treatment for nephrolithiasis or urolithiasis, and not further specified bladder or prostate surgeries.

^‘Other’ included procedures from subcategories comprising <5% of the study population, including skin (e.g. abscess drainage, wound debridement), gynaecological(e.g. hysterectomy, tubectomy, prolapse surgery), transplantation, endocrine (e.g. (para/hemi)thyroidectomy, parotidectomy), lung, and reconstructive surgery (e.g. breast reconstruction or reduction, revision of amputation stump).

**Table S7: Primary outcomes in subgroups of type of surgery.**

| **Type of surgery** | Worst pain | Movement-evoked pain | Pain at rest | Current anxiety | Worst anxiety | Total stress score | Sleep quality* |
| --- | --- | --- | --- | --- | --- | --- | --- |
| - Gastro-intestinal (N = 230) | 7 (4-8) | 6 (3-7) | 4 (2-6) | 10 (0-31) | 23.5 (0-60) | 12 (7.5-16) | 3.93 (2.82-5.03) |
| - Vascular (N = 116) | 5 (2-8) | 4 (2-7) | 3 (1-6) | 0 (0-30) | 0 (0-57) | 12 (8-16) | 1.74 (0.20-3.28) |
| - Trauma (N = 91) | 7 (5-9) | 6 (4-8) | 5 (2-7) | 20 (0-50) | 35 (0-70) | 11 (7-16) | 4.20 (2.65-5.74) |
| - Oncological (N = 63) | 5 (3-7) | 4 (2-7) | 3 (1-5) | 9 (0-40) | 10 (0-50) | 12 (8-15) | 3.95 (2.01-5.88) |
| - Urological (N = 54) | 4 (1.75-7) | 3 (1-5) | 3 (1-4) | 0 (0-30) | 15.5 (0-50.25) | 9 (4.75-15) | 4.66 (2.57-6.75) |
| - Skin (N = 23) | 4 (1-8) | 2 (1-5) | 2 (0-4) | 0 (0-20) | 13 (0-53) | 9 (4-14) | 1.74 (-1.80-5.28) |
| - Gynaecological (N = 16) | 3 (0-7) | 3 (0.5-6.75) | 1.5 (0-4.5) | 10 (0-51.5) | 22.5 (0-77.5) | 9 (5-12) | 2.0 (-1.09-5.09) |
| - Transplantation (N = 11) | 4 (1-7) | 4 (0-8) | 4 (0-5) | 10 (0-20) | 10 (0-60) | 10 (5-14) | 4.18 (-2.90-11.27) |
| - Endocrine (N = 10) | 2.5 (1-6.5) | 2.5 (1-4.5) | 2 (1-3) | 4 (0-15) | 10 (0-37.5) | 11.5 (8-16.5) | 3.0 (-3.03-9.03) |
| - Lung (N = 8) | 4 (1.25-6.75) | 4 (0.25-6.5) | 3 (1-4.75) | 0 (0-4.5) | 0 (0-27.5) | 9 (7-19) | 3.50 (-2.50-9.50) |
| - Reconstructive (N = 7) | 1 (0-7) | 2 (0-3) | 1 (0-1) | 0 (0-29) | 0 (0-40) | 9 (5-8) | 2.0 (-3.34-7.34) |
| - No surgical procedure (N = 77) | 7 (3-8) | 6 (2-8) | 4 (2-7) | 0 (0-40.5) | 20 (0-49.5) | 13 (8-17) | 6.41 (4.50-8.32) |
| - Other (N = 22) | 4.5 (1.75-8) | 4 (3-6.25) | 4 (1-5.25) | 24.5 (0-62.5) | 27.5 (0-72.5) | 13 (7.5-17) | 1.35 (-1.45-1.58) |

Values are median (interquartile range) unless otherwise indicated.

* Calculated as the mean difference between home and hospital summary score (95% confidence interval
